# Distraction osteogenesis and arthrodesis as a new surgical option for chondrosarcoma in the distal tibia

**DOI:** 10.1186/s12957-015-0604-8

**Published:** 2015-05-22

**Authors:** Zhengxiao Ouyang, Xuezheng Xu, Linqing Li, Yi Luo, Jianfan Liu, Xin Wang, Xinyu Yao, Gang Huang, Xianan Li

**Affiliations:** Department of Orthopaedics, Hunan Cancer Hospital and The Affiliated Cancer Hospital of Xiangya School of Medicine, Central South University, 283 Tongzipo Road, Changsha, Hunan 410012 China

**Keywords:** Limb salvage, Chondrosarcoma, Distraction osteogenesis, Bone lengthening, Arthrodesis

## Abstract

Recent advances in the management of bone tumors have led to a significant increase in the survival rates of patients with malignant bone tumors. Thus, limb salvage surgery has gained importance for preserving limb function in the management of bone tumors. However, surgery presents unique difficulties in terms of the biomechanics and obtaining a soft-tissue cover, such as when the ankle is involved in the primary malignant bone tumor. We report a case of chondrosarcoma of the distal tibia treated with wide en bloc resection arthrodesis and reconstruction of the defect using distraction osteogenesis, which offers an effective alternative protocol for limb salvage. The patient has remained disease free for 3 years since the initial surgery and can maintain normal limb athletic function.

## Background

Recent advances in the management of bone tumors, including preoperative radiological evaluation, chemotherapy, materials and implant technology, and surgical technique, have led to a significant increase in the survival rates of patients with malignant bone tumors [1–3]. Therefore, limb salvage surgery has gained importance for providing adequate tumor resection while preserving a functional limb [2, 4, 5]. Various methods, including biological (e.g., vascularized autograft, allograft, recycled bone treated by radiation, autoclaving, pasteurization, liquid nitrogen, and distraction osteogenesis) and non-biological (prosthesis) methods, have been established for the reconstruction of bone defects after malignant or benign bone tumor excision; however, a gold standard method for reconstruction does not exist [2, 3, 6], and there are few reports of reconstruction of large bone defects in the distal tibia following wide resection of malignant bone tumors, adding to the challenge of limb salvage surgery.

This report describes a patient with chondrosarcoma of the distal tibia who underwent limb salvage surgical resection followed by limb lengthening and arthrodesis. There are no reports of the use of this combined approach to produce limb salvage and a functional lower extremity. The patient received a detailed explanation concerning the surgical procedure and the intent to submit data from the case for publication, and he provided consent.

## Case presentation

**Fig. 1:**
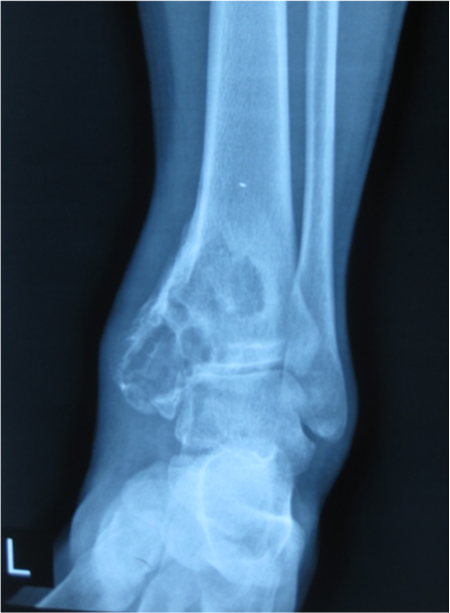
Radiographs revealing an aggressive lesion of the distal tibia

**Fig. 2:**
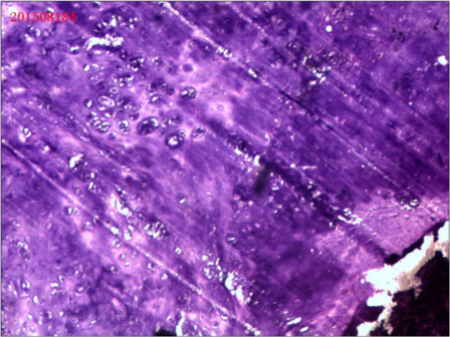
Photomicrographs of the lesion

**Fig. 3:**
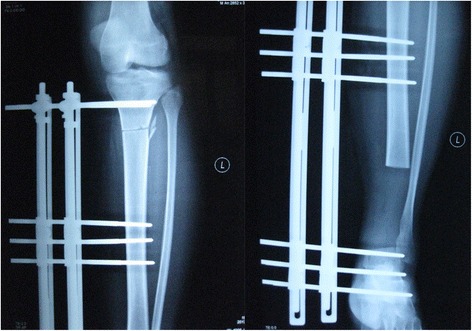
Distal tibia lengthening with a unilateral external fixator after excision of the tumor

**Fig. 4:**
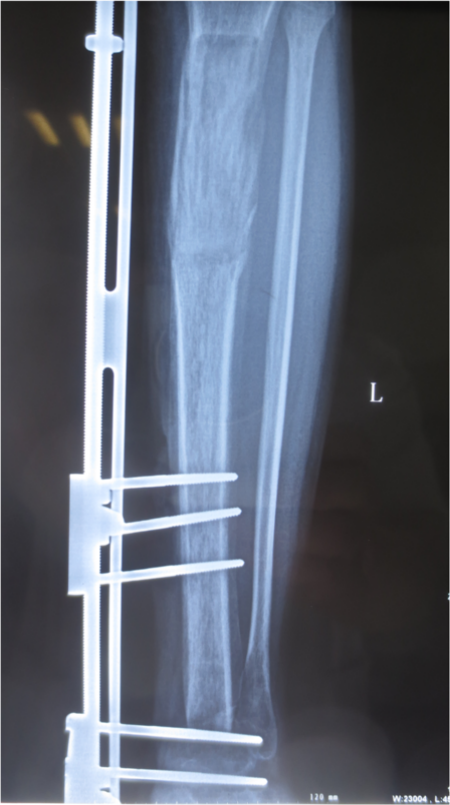
Ankle joint arthrodesis after bone lengthening

**Fig. 5:**
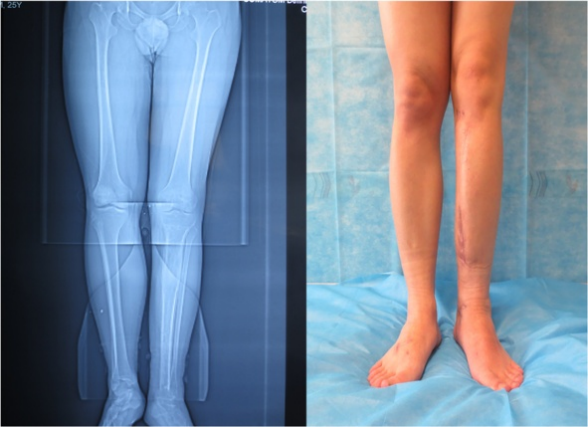
Nine months after surgery, the affected limb was similar to the unaffected side in length, form, and function

A 23-year-old man presented with left leg pain. Radiographs revealed an aggressive lesion of the distal tibia (Fig. 1). Biopsy (Fig. 2) and appropriate staging studies confirmed stage IIB chondrosarcoma [7]. He was treated with wide en bloc resection, including the distal third of the tibia and the cartilage of the talus in the tibiotalar joint. He then underwent a distal tibia lengthening with a unilateral external fixator (Fig. 3). Gradual distraction started 5–7 days after surgery and was applied at a rate of 1 mm per 36 hours, and 8 cm of length was gained and corticalized over a 6-month period. Fusion of the tibiotalar joint was then allowed to proceed for 3 months before removal of external fixation (Fig. 4). The current limb-length discrepancy is minimal (Fig. 5). He has remained disease free for 3 years since the initial surgery and can maintain normal limb athletic function.

The *external fixation index* calculated by dividing the entire duration of external fixation by the length of bone regeneration was 36 days/cm; the *distraction index* calculated by dividing the duration of distraction by the length of bone regeneration was 11.08 days/cm, and the *maturation index* calculated by dividing the duration of external fixation by the length of bone regeneration was 25.8 days/cm, as measured from the completion of distraction to the removal of external fixation. The patient was evaluated via physical examinations and radiographs throughout the follow-up period. The function of the affected limb was assessed according to the revised 30-point functional classification system established by the International Society of Limb Salvage and the Musculoskeletal Tumor Society (MSTS) [7]. The MSTS score was 94 at the final follow-up, and the Foot and Ankle Disability Index (FADI) for patients with a tumor around the ankle joint was 90.

## Discussion

Limb salvage surgery is currently a very commonly performed procedure [8]. However, restoration and long-term maintenance of good limb function after resection of a bone tumor remain a challenge. The ideal reconstruction should have biological affinity and infection resistance. More importantly, for weight-bearing lower extremities, sufficient biomechanical strength and durability are indispensable [9–11]. At present, there is no gold standard method for reconstruction [2, 3, 6], and there are few reports of reconstruction of large bone defects in the distal tibia following wide resection of malignant bone tumors, adding to the challenge of limb salvage surgery.

After considering the difficulties in determining the best treatment strategy for the current patient, another hospital suggested amputation after open biopsy for the following reasons: (1) amputation has been the standard surgical treatment, with satisfactory functional results using an appropriate prosthesis [12], and (2) difficulties exist both in terms of the biomechanics and obtaining a soft-tissue cover [13]. For such a young patient, return to function and preservation of cosmetic appearance are of great importance, especially considering his need to make social and financial plans for the prolonged rehabilitation period. Furthermore, patients treated with limb salvage surgery for distal lower leg sarcoma had excellent final functional results without impairing the oncologic results [14]. This is also favorable because (1) there are fewer musculotendinous attachments in the distal tibia than elsewhere, and (2) these attachments often contribute to the tumor breaking through the compartment.

Thus, we decided to perform limb salvage surgery. In this case, the tumor extended into the meta-epiphysis; thus, intra-articular resection including the articular surface was required. Under these circumstances, reconstructive options present unique difficulties. Endoprosthetic replacement has been reported to possess many advantages, including early stability, mobilization, weight-bearing, and rapid restoration of function with a good functional outcome; however, problems such as infection, mechanical failure, and aseptic loosening exist and may limit the long-term survival of the prosthesis, increasing the risk of revision over time [12, 15]. In addition, the lack of soft tissue after wide resection might add to the risk of infection and ultimately amputation. For allografts, there are high rates of complications such as nonunion [16], infection, fracture, degeneration of the articular surface, graft resorption, joint instability, and pathological fractures [16–19]; accordingly, allografts are considered a temporary solution in the management of malignant bone tumors [20]. Moreover, postoperative chemotherapy delays incorporation and union because of negative effects on healing and revascularization [21]. Generally, complications gradually increase over time in limbs reconstructed with tumor prostheses or allografts, and limb function also worsens.

Distraction osteogenesis has been widely used as a biological approach for repairing segmental bone defects [22, 23] and can regenerate living bone of sufficient strength; thus, it can preserve limb function over a lifetime [24, 25]. Excellent results have been reported [1, 26–29] for distraction osteogenesis in reconstruction after massive bone loss due to tumor resection, and it has been concluded to be beneficial in patients with an expectation of long-term survival. The indications for bone distraction are (1) stage IIB malignant bone tumors when chemotherapy is judged to be effective and an epiphysis could be preserved or (2) low-grade or aggressive benign bone tumors [1]. However, none of these previous studies reported results when the ankle was involved. In the ankle, good functional and oncological results have been reported for arthrodeses with autogenous fibular strut grafts stabilized using an Ilizarov external fixator [30] and arthrodeses with autogenous bone grafts [13, 31, 32]. However, the period for graft union is long, especially in patients with non-vascularized grafts (18 months) [30].

In our opinion, there are at least five reasons for choosing distraction osteogenesis and joint fusion for patients with malignant tumors in the distal tibia. First, retaining most of the original tibia and sufficient epiphysis thickness permits full weight bearing. Second, patients with malignant bone tumors in such locations have a favorable prognosis [14]. Third, the same amount of bone ossification as seen in distraction osteogenesis during reconstruction has not been observed in vascularized fibulae [26]. Fourth, the need for bone banking, donor site morbidity, and the risk of disease transmission associated with allografts are eliminated [33]. Moreover, our combination of surgical options prevented the patient from walking for just 9 months.

Disadvantages in bone distraction include delayed union at the docking site and pin- or wire-tract infection. However, such complications were not observed in this case. Once function has been restored, it can be maintained throughout life, without anxiety concerning loosening or revision. The use of distraction osteogenesis in the treatment of infection and tumors is well established but has not been reported in treating severe bone loss after tumor resection at the ankle region. Here, the indication of bone distraction was extended to include such a case in which the epiphysis could not be preserved.

Several points should be noted in the application of such surgical procedures. (1) There is an abrupt increase in the complication rate during bone lengthening of the tibia in patients with bone defects ≥15 cm after tumor excision; this is due to the greater length of time required [34, 35]. Some researchers have recommended that such cases should be excluded [36]. In contrast, another group reported different results [26]. (2) Charnley was the first to describe compression ankle arthrodesis using a uniplanar external fixator. Since then, many types of fixators have evolved to improve fixation stability [37], including the excellent representative Ilizarov apparatus. However, the choice of technology depends on many factors such as the surgeon’s familiarity and the economic status of the institution. (3) Our patient was not simultaneously undergoing chemotherapy during the distraction period, so we are unable to comment on the use of distraction osteogenesis with these treatments. However, it is reported that chemotherapy has no hazardous effect on bone regeneration with distraction osteogenesis [26, 38] if regional blood flow is maintained within the normal range.

## Conclusions

In general, the choice of reconstructive procedure should be considered with several factors, such as the site and involvement of the tumor, pathology and biological behavior of the tumor, life expectancy, and predicted function of the limb. We believe that wide en bloc resection arthrodesis, with reconstruction of the defect using distraction osteogenesis, offers an effective alternative protocol for limb salvage in cases of chondrosarcoma of the distal tibia. Though reconstruction using bone distraction requires both time and effort, it can provide excellent long-term outcomes, resulting in a stable reconstruction that functionally restores the natural limb and is cosmetically appealing.

## Consent

Written informed consent was obtained from the patient for publication of this case report and any accompanying images. A copy of the written consent is available for review by the editor-in-chief of this journal.
